# MITOCHONDRIAL CHAPERONES MAY DICTATE OUTCOMES IN WOMEN OF ADVANCED MATERNAL AGE

**DOI:** 10.1093/geroni/igae098.2258

**Published:** 2024-12-31

**Authors:** Hannah Archer, Caroline Smith, Arthur Chang, Renee Chosed

**Affiliations:** Greenville School of Medicine, Greenville, South Carolina, United States; North Greenville University, North Greenville University, South Carolina, United States; UT Health, Houston, Texas, United States; Greenville School of Medicine, Greenville, South Carolina, United States

## Abstract

**Background:**

A patient may seek the use of IVF technology when struggling to become pregnant. To improve IVF outcomes, research focuses on identifying markers to allow embryologists to transfer embryos with the highest chance of a successful pregnancy. RNASeq analysis indicated that DNAJC19 gene expression was higher in embryos that resulted in successful implantation. DNAJC19 is a mitochondrial chaperone whose role is to provide instructions for producing proteins within the mitochondria. Our study analyzes DNAJC19 expression in patients of advanced maternal age (>35 years) to identify an additional biomarker during preimplantation embryo development to select the most viable embryo for successful implantation.

**Methods:**

Blastocoel fluid-conditioned media was collected from day-5 IVF-embryos that underwent PGT-A and were from mothers of advanced maternal age. RNA was purified from pooled blastocoel fluid samples, and then cDNA was synthesized. RT-qPCR assessed 18S (control gene) and DNAJC19 levels.

**Results:**

Expression of DNAJC19 was higher blastocoel fluid samples from euploid embryos from mothers of advanced maternal age when compared to aneuploid embryos from mothers of advanced maternal age. Conclusions: DNAJC19 is an important mitochondrial chaperone that assists in protein import in the mitochondria. This preliminary analysis indicates a higher expression of DNAJC19 in blastocoel fluid from embryos from mothers of advanced maternal age. This finding suggests that mitochondrial function is elevated in euploid embryos from mothers over 35 years old. Further research is needed to determine the significance of this observation in regards to embryo implantation.

